# Shortened Hospital Stay and Improvement of Inpatient Management for Decompensated Heart Failure Patients

**DOI:** 10.7759/cureus.90347

**Published:** 2025-08-17

**Authors:** Md Khalilur Rahman, MD Anik Rahman, Masuma Akter, Mohammad Fahad Chowdhury

**Affiliations:** 1 Acute Medicine, Huddersfield Royal Infirmary, Huddersfield, GBR; 2 Acute Medicine, Calderdale and Huddersfield NHS Foundation Trust, Huddersfield, GBR; 3 General Medicine, Calderdale and Huddersfield NHS Foundation Trust, Huddersfield, GBR

**Keywords:** decompensated heart failure, good quality of life, intravenous diuretics, reduced risk of complications, shortened hospital stay

## Abstract

Heart failure constitutes a pandemic health condition that is distinguished by its association with high morbidity and mortality statistics. Acute heart failure episodes necessitating hospital admission are characterised by poor clinical outcomes and increased hospitalisation duration, particularly within the context of an ageing demographic. While standard therapeutic regimens encompass IV diuretics, ARNIs (angiotensin II receptor blockers and a neprilysin inhibitor), beta-blockers, mineralocorticoid receptor antagonists, and SGLT2 (sodium-glucose cotransporter-2) inhibitors, insufficient monitoring and modification of clinical and biochemical indicators contribute to extended hospital stays and increased adverse event rates.

## Introduction

Acute decompensated heart failure is a prevalent cause of hospital admissions, associated with significant morbidity and death rates [1]. Acute heart failure events requiring hospital admission are marked by unfavourable clinical outcomes and prolonged hospitalisation, especially in an elderly population. Intravenous diuretic therapy constitutes the primary element of treatment [2]. This treatment requires meticulous titration and is linked to several consequences, including acute kidney injury (AKI), hypovolemia, ototoxicity, and electrolyte imbalance [3]. Consequently, systematic monitoring of patients on intravenous diuretic therapy is essential to evaluate therapeutic outcomes, adjust treatment protocols, and proactively identify and address complications. At the ward level, the suggested monitoring entails regular weight assessments, electrolyte evaluations, and input/output documentation [4]. Nonetheless, compliance with this requirement is not consistently attained due to competing clinical demands in the wards [5]. Inadequate monitoring of clinical and biochemical markers, despite the application of guideline-directed medical therapy (IV diuretics, ARNIs, beta-blockers, MRAs, and SGLT2 inhibitors), is significantly associated with extended hospital stays and increased rates of complications [3].

## Materials and methods

A retrospective review was conducted on electronic patient records from 50 individuals in the Elderly and General Medical wards. The bicyclic investigation of subjects with acute heart failure decompensation implemented comprehensive surveillance protocols featuring daily weight documentation, fluid equilibrium parameters, serial biochemical analysis of renal markers and electrolytes, and systematic fluid intake/output recording. The research focused on the emergence of complications, length of hospital stays, and adherence to monitoring protocols alongside standard care pathways. The initial cycle enrolment criteria encompassed patients admitted to general medical or frailty wards who received intravenous diuretic therapy for at least three days. A comprehensive review of medical documentation was conducted for the qualified patient population to obtain data concerning the degree of clinical supervision and the prevalence of adverse outcomes. Complications encompassed AKI, characterised by an increase in creatinine above the normal limit, and electrolyte imbalance, which involved sodium levels outside the range of 130-150 mmol/L and potassium levels outside the range of 3-5.5 mmol/L. Concurrently, we obtained perspectives from a representative sample of physicians and registered nurses regarding barriers that impede optimal monitoring protocols for heart failure patients. Subsequent to the initial data collection phase, we launched our intervention by installing poster displays that reinforced the clinical standards expected in acute decompensated heart failure care. The project team conducted instructional seminars for certain wards. The subsequent data collection phase employed comparable enrolment criteria for the second cohort.

## Results

**Figure 1:**
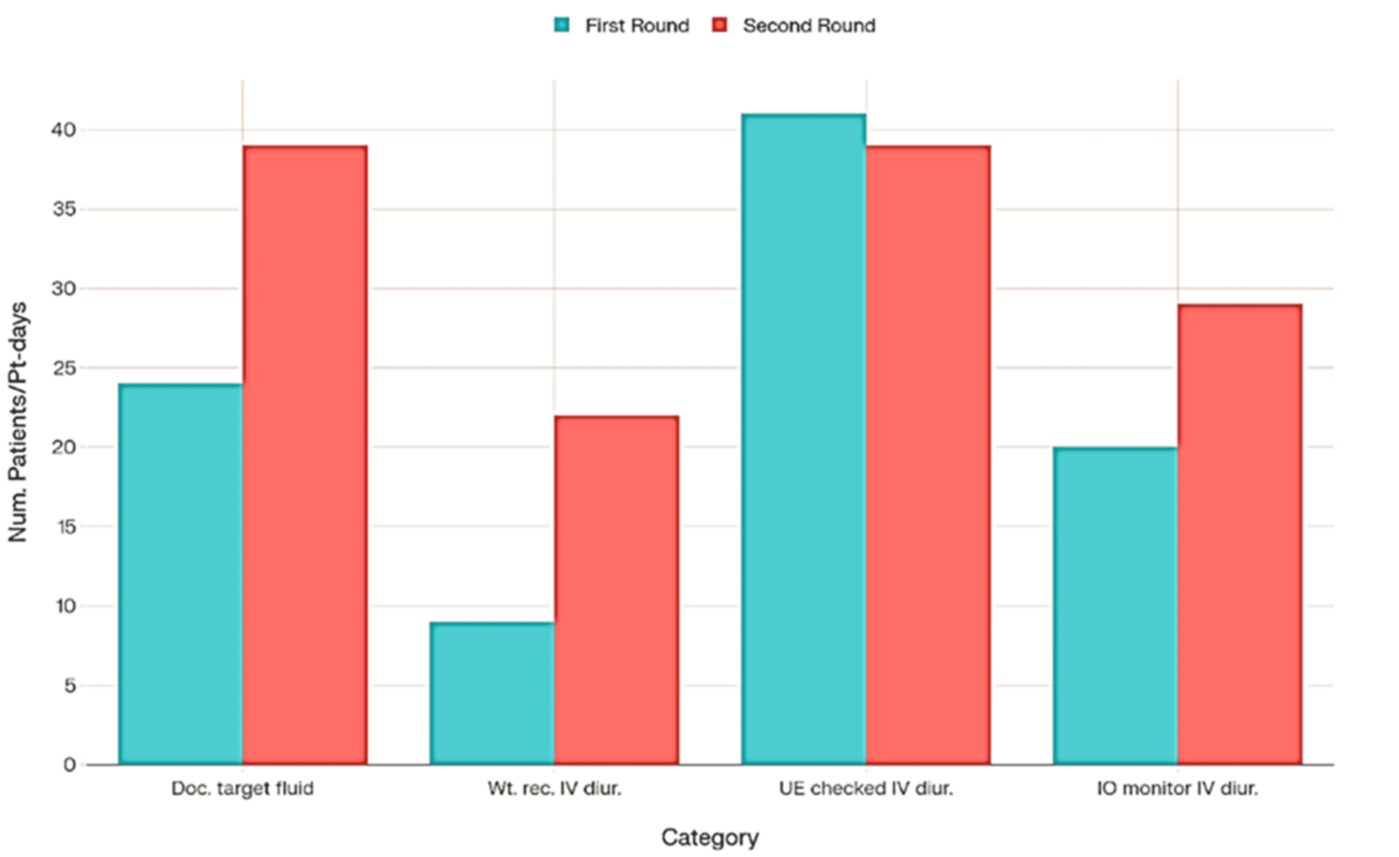
Daily fluid charting, weighing, UEs, and Intake-output monitoring comparison between the two QIP cycles Doc.: Documented; Wt. rec. IV diur.: Weight recorded during Intravenous Diuretics; QIP: Quality Improvement Project, UEs: Urea and Electrolytes, IO: Intake-output monitoring

Intravenous diuretic medication was delivered to 50 patients meeting the inclusion criteria, with a total treatment length of 425 days and a median individual treatment period of 8.5 days. In the initial data analysis, approximately 24 out of 50 patients had a documented target fluid balance or fluid restriction, compared to 39 out of 50 patients in the second round (Figure 1). During the first round of intravenous diuretic therapy, weight was recorded for 9 out of 50 patient-days, urea and electrolytes were assessed for 41 out of 50 patient-days, and input/output monitoring was documented for 20 out of 50 patient-days (Figure 1). In the second round, weight was recorded for 22 out of 50 patient-days, urea and electrolyte assessments were performed on 39 out of 50 patient-days, and input/output charting was done for 29 out of 50 patient-days (Figure 1).

**Figure 2:**
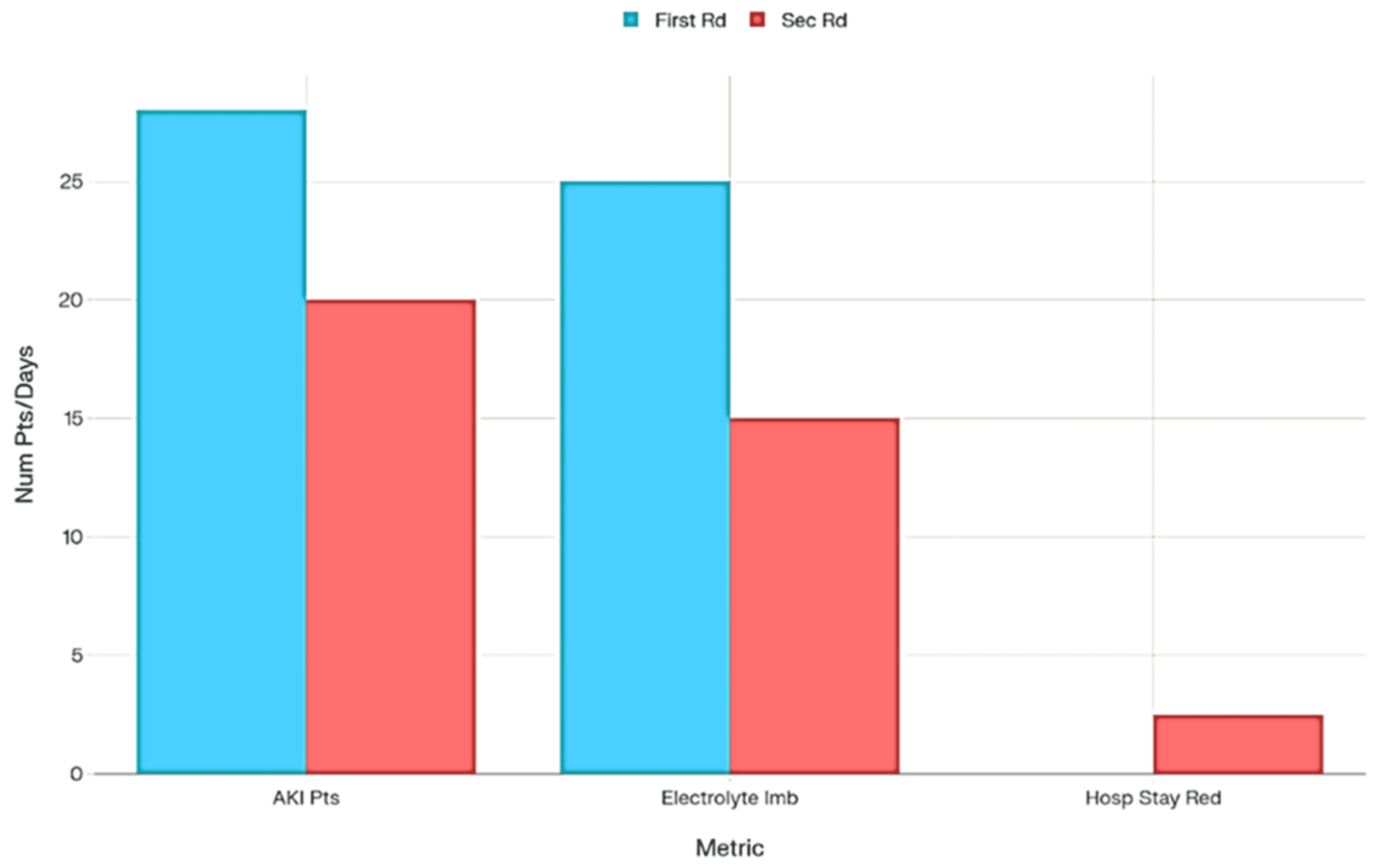
Comparison of AKI incidence, electrolyte imbalance, and hospital stay reduction between first and second rounds during intravenous diuretic therapy AKI: acute kidney injury; Pts: patients; Imb: imbalance; Hosp stay red: hospital stay reduction

During intravenous diuretic therapy, 28 out of 50 patients (rounded from 27.5) in the initial round experienced AKI as identified by the EPR AKI alert. This number decreased to 20 out of 50 patients in the subsequent round (Figure 2). The proportion of patients with electrolyte imbalances decreased from 50% (25/50) before the intervention to 30% (15/50) afterwards, using the same criteria for potassium and sodium levels (Figure 2). The second cycle showed a reduction of approximately 2-3 days in hospital stay duration compared to the initial round (Figure 2).

This quality improvement project (QIP) demonstrates measurable advances in clinical outcomes for patients with decompensated heart failure, specifically focusing on the incidence of AKI and the reduced time to become medically optimised for discharge (MOFD) across two consecutive QIP cycles.

Reduction in the incidence of acute kidney injury (AKI)

**Figure 3:**
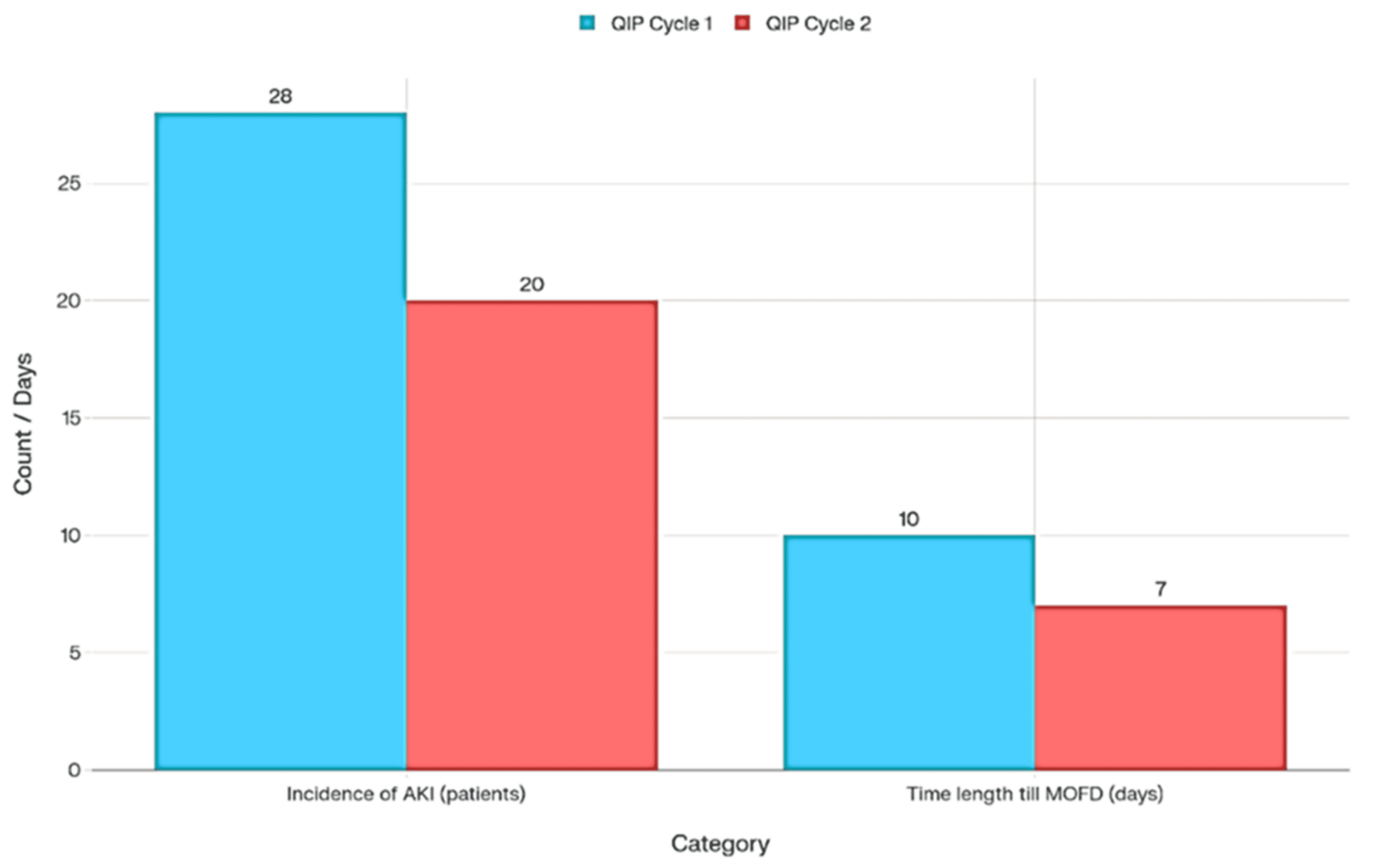
Incidence of AKI and time length till MOFD, comparison between the two QIP cycles AKI: acute kidney injury; MOFD: Medically Optimised for Discharge; QIP: Quality Improvement Project

The data show a marked reduction in AKI incidence, declining from 28 patients in QIP cycle-1 to 20 patients in QIP cycle-2, a drop from 55% to 40% of the cohort (Figure 3). This improvement suggests that the implemented interventions, such as stricter fluid balance management, enhanced monitoring protocols, and earlier recognition and treatment of renal dysfunction, had a significant positive effect. AKI is a common and serious complication among heart failure patients on intravenous diuretics, associated with increased morbidity, prolonged hospitalisation, and greater resource utilisation. By decreasing AKI rates, the QIP initiative directly contributes to better patient safety and potentially reduces overall healthcare costs.

Shorter time to become medically optimised for discharge (MOFD)

The duration until MOFD also improved, falling from 10 days in QIP cycle-1 to 7 days in Cycle-2 (Figure 3). While a reduction in time to MOFD may at first appear detrimental, in this context, it likely indicates that patients at risk were identified and managed more aggressively, possibly leading to either faster recovery or swifter escalation of care.

Impact of interventions

The improvement across both metrics can be attributed to several targeted interventions introduced between cycles, as discussed in earlier project phases.

Enhanced Documentation and Monitoring

There was greater adherence to best practices, with more patients receiving documented target fluid balance or fluid restriction and more frequent input/output and electrolyte monitoring.

Education and Feedback

Staff training and feedback on heart failure, AKI risks, and organ dysfunction signs likely improved clinical alertness and response times.

Structured Protocols

Clearer and more standardised protocols for intravenous diuretic therapy helped reduce variability in care, facilitating earlier intervention and tighter control of risk factors. These findings underline the value of iterative QIP cycles and multi-disciplinary engagement in hospital practice. Achieving sustainable reductions in AKI and optimising MOFD timelines not only improves patient outcomes but may also contribute to shortened hospital stays, lower readmission rates, and fewer complications. This cycle-wise improvement illustrates that simple changes in practice, when rigorously implemented and monitored, can yield substantial benefits.

## Discussion

This study demonstrates the significant influence of organised hospital monitoring and holistic treatment approaches on patient outcomes in acute decompensated heart failure cases. Consistent with previous literature [1-2], our findings demonstrate that hospital stays are prolonged and clinical outcomes often worsen in the absence of robust clinical and biochemical surveillance, even when guideline-directed therapies are appropriately employed.

Findings confirm that thorough education of clinical personnel regarding heart failure management principles, specifically early recognition and mitigation of risks such as AKI, is fundamental to improving patient outcomes and decreasing hospital length of stay. Enhanced vigilance, regular weight and electrolyte monitoring, and diligent input/output tracking following staff training were associated with earlier identification of clinical deterioration, allowing for prompt interventions and potentially forestalling multi-organ failure development.

This improvement aligns with the evidence cited by Zhang et al. 2024, highlighting that overcoming real-world implementation challenges for guideline-directed medical therapy can maximise benefits for heart failure patients [5]. Our data substantiate that compliance with recommended monitoring strategies remains a significant hurdle, often due to competing demands in busy hospital environments [5]. Addressing these barriers through focused staff education and feedback mechanisms directly contributed to improved outcomes within our patient cohort.

Furthermore, the reduction in AKI incidence and shorter time to become MOFD in the latter QIP cycle reinforces the necessity of integrating regular training and feedback loops in heart failure management protocols. These interventions not only optimise the use of core pharmacological agents such as IV diuretics, ARNIs, beta-blockers, MRAs, and SGLT2 inhibitors, but also support individualised patient care and responsiveness to early warning signs.

Despite these positive outcomes, several limitations warrant consideration. The study may be limited by its scope and sample size, and the generalisability of results may be influenced by institution-specific factors such as staffing ratios and resource availability. Additionally, while targeted monitoring improved short-term outcomes, longer-term follow-up is needed to determine sustained benefits and readmission rates.

In summary, our study supports the proposition that improving clinical and biochemical monitoring, alongside ongoing staff education, is essential for enhancing the care and reducing the hospital stay of patients with decompensated heart failure. Future research should focus on multicentre trials and investigating technology-driven monitoring solutions to further standardise and optimise inpatient heart failure management.

## Conclusions

Following strict monitoring protocols reduced complications and shortened hospital stays for decompensated heart failure patients. Our trial revealed that this patient cohort was inadequately monitored. Insufficient staff awareness, increased workload constraints in healthcare settings, and accelerated patient discharge patterns were among the obstacles that contributed to these findings.

Specific standard metrics were enhanced following a little awareness effort. There remains potential for advancement in several domains. It is unlikely that educational programmes alone will provide comprehensive solutions to all identified challenges. Ongoing advancement is expected to arise from further phases of research employing interventions such as electronic dashboard systems, streamlined procedures, and enhancements in monitoring efficiency.
